# Supplementary material for: Kinetic Investigations on the Chiral Induction by Amino Acids in Porphyrin J-Aggregates
Source: Int J Mol Sci. 2023 Jan 15;24(2):1695. doi: 10.3390/ijms24021695 (PMC9860692; doi:10.3390/ijms24021695)
Supplement: Supplementary file 1 [file ijms-24-01695-s001.zip › ijms-2148469-supplementary.pdf]

Supporting Information

for

Kinetic Investigations on the Chiral Induction by Amino acids in Porphyrin J-Aggregates

Roberto Zagami,[1] Maria Angela Castriciano,[1] Andrea Romeo,[1,2] and Luigi Monsù Scolaro*[1,2]

Table S1. Structural formulas, abbreviations and isoelectric points (IEP) of the amino acids

Figure S1. Bar graph of the values of *m*

Figure S2. Bar graph of the values of *n*

Figure S3. Bar graph of the values of the extinction values at equilibrium and their percent deviations.

Table S1. Structural formulas, abbreviations and isoelectric points (IEP) of the amino acids used in the present investigations.

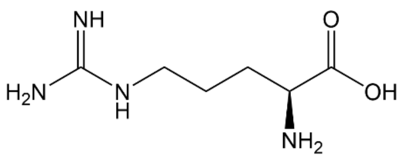	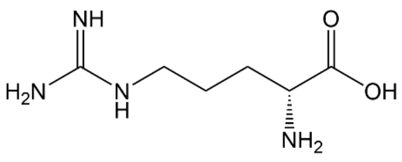	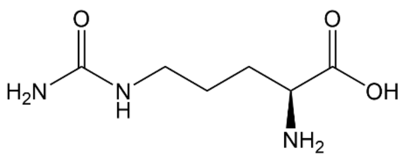
L-ARGININE (**L-Arg**; IEP 10.76)	D-ARGININE (**D-Arg**; IEP 10.76)	L-CITRULLINE (**L-Cit**; IEP 6.05)
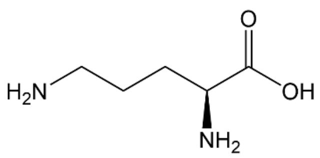	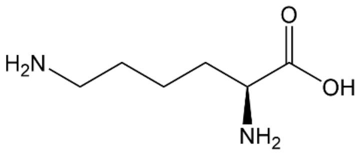	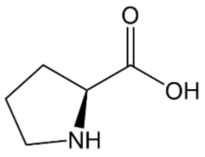
L-ORNITHINE (**L-Orn**; IEP 9.75)	L-LYSINE (**L-Lys**; IEP 9.47)	L-PROLINE (**L-Prol**; IEP 6.30)
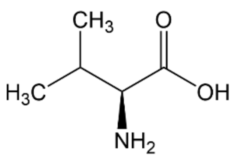	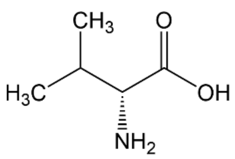	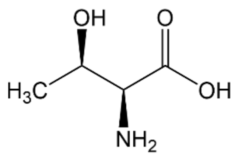
L-VALINE (**L-Val**; IEP 5.96)	D-VALINE (**D-Val**; IEP 5.96)	L-THREONINE (**L-Thr**; IEP 5.50)
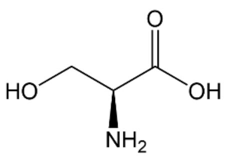	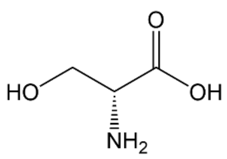	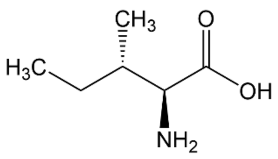
L-SERINE (**L-Ser**; IEP 5.68)	D-SERINE (**D-Ser**; IEP 5.68)	L-ISOLEUCINE (**L-Ile**; IEP 6.02)
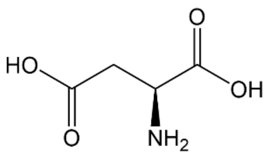	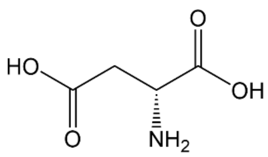	
L- ASPARTIC ACID (**L-Asp**; IEP 2.77)	D- ASPARTIC ACID (**D-Asp**; IEP 2.77)	

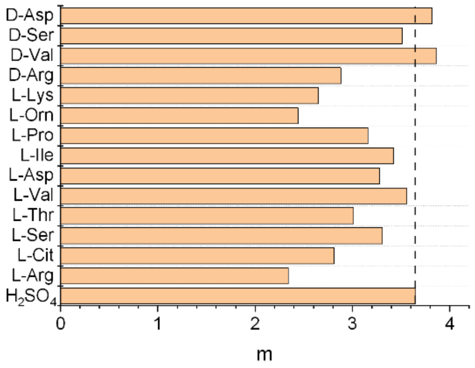

Figure S1. Bar graph of the values of *m* for the aggregation of $TPPS_4$ induced by H_2SO_4 in the presence of amino acids (the dashed black line mark the reference value obtained in the absence of added amino acids).

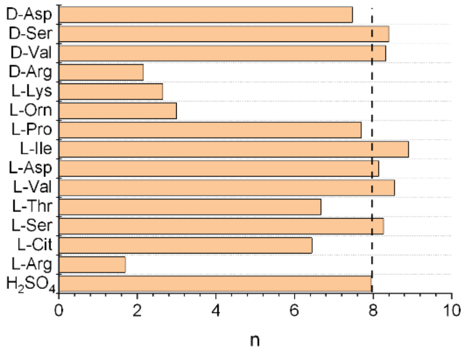

Figure S2. Bar graph of the values of *n* for the aggregation of $TPPS_4$ induced by H_2SO_4 in the presence of amino acids (the dashed black line mark the reference value obtained in the absence of added amino acids).

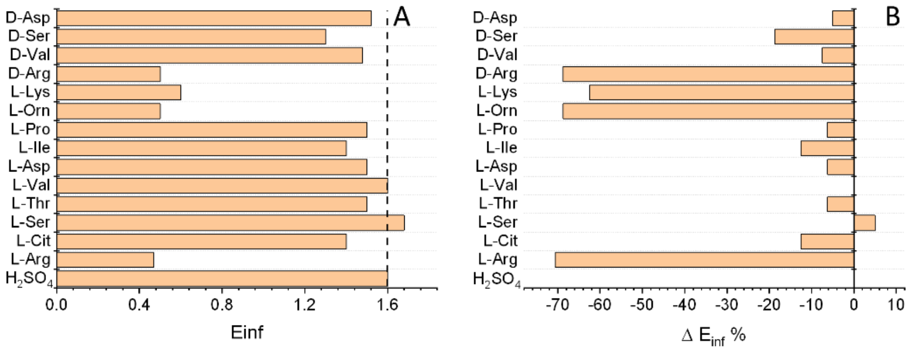

Figure S3. Bar graph of (A) the values of the extinction values at equilibrium for the aggregation of $TPPS_4$ induced by H_2SO_4 in the presence of amino acids (the dashed black line mark the reference value obtained in the absence of added amino acids); (B) the percent deviation of extinction values at equilibrium with respect to the reference value measured in the presence of H_2SO_4 only.
